# Metabolic profiles of amino acids in patients with crohn’s disease-associated perianal fistulas and cryptoglandular anal fistulas

**DOI:** 10.1038/s41598-025-33334-7

**Published:** 2026-01-19

**Authors:** Kai Ma, Cong Hu, Yi Fu, Yu Liu, Feiyang Weng, Yibo Yao, Chen Wang

**Affiliations:** 1 Department of Proctology, Longhua Hospital, Shanghai University of Traditional Chinese Medicine, Shanghai, 200032 China; 2 Institute of Interdisciplinary Integrative Medicine Research, Shanghai University of Traditional Chinese Medicine, Shanghai, 201203 China; 3 Key Laboratory of Basic Pharmacology of Ministry of Education and Joint International Research Laboratory of Ethnomedicine of Ministry of Education, Zunyi Medical University, Zunyi, 563000 China

**Keywords:** Amino acid, Crohn’s disease-associated perianal fistula, Cryptoglandular anal fistulas, Biomarker, Biomarkers, Diseases, Gastroenterology, Medical research

## Abstract

Crohn’s disease-associated perianal fistulas (PFCD) and Cryptoglandular anal fistulas (CAF) are two types of fistulas with similar clinical manifestations but distinct etiologies and therapeutic strategies. This study aims to investigate serum amino acid metabolic profiles in patients with CAF and PFCD based on UPLC-MS/MS. Serum samples were enrolled from newly diagnosed and treatment-naïve 36 patients with active PFCD and 36 patients with CAF as controls. Clinical characteristics were systematically documented. Serum concentrations of 25 amino acids were quantitatively determined using UPLC-MS/MS with isotopically labeled internal standards. Multivariate statistical analyses were employed to discriminate metabolic profiles between groups. Pathway enrichment analysis of differential amino acids was performed using Metabo Analyst 5.0. The PFCD cohort exhibited significantly lower total serum amino acid concentrations compared to CAF group, with 13 differentially expressed amino acids identified. Multivariate analyses revealed a significant separation trend between the two groups, eight amino acids were prioritized as candidate diagnostic biomarkers. Metabolic pathway enrichment analysis demonstrated that five pathways showed significant associations with metabolic disparities between the two groups. The proposed biomarker panel may assist in differentiating PFCD from CAF and guide individualized therapy.

**Supplementary Information:**

The online version contains supplementary material available at 10.1038/s41598-025-33334-7.

Perianal fistula is a common medical and surgical problem, resulting in epithelial-lined tract connecting the rectum or anal canal to the perianal exterior skin surface^1^. In Europe, according to population-based inception cohorts, the data concluded that the incidence of perianal fistulas is reported to be around 2.6–3.8 per 10,0000 people, with a peak incidence between the ages of 20 and 40 years, and it is more commonly observed in men than in women^2,3^. The pathogenesis of perianal fistulas formation is not yet fully understood. The vast majority (> 90%) of perianal fistulas are classified as cryptoglandular anal fistulas (CAF), the prevailing perspective on CAF attributes their origin to inflammation of the anal glands^4^. The second most common type of perianal fistulas are those associated with Crohn’s disease-associated perianal fistulas (PFCD), notably, complex fistulas are more frequently encountered in patients with Crohn’s disease^5,6^. Patients with Perianal fistulas are often characterized with significant clinical manifestations, the characteristic symptoms-anorectal pain, purulent discharge, recurrent abscess formation, and potential fecal incontinence-often profoundly compromise patients’ quality of life and impose a considerable economic burden to those patients^7^.

Discriminating PFCD from CAF remains a key diagnostic obstacle in perianal disease. Although intestinal inflammation is typically associated with PFCD, endoscopic activity is absent in 10%-15% of patients during initial presentation. Importantly, perianal fistulas serve as the initial symptom complex of CD in approximately 10% of patients, often leading to substantial delays in definitive diagnosis^8,9^. The treatments of these two groups of fistulas are significantly different, CAF are mainly treated with surgery, however, in PFCD, there is extensive evidence supporting a multidisciplinary approach, encompassing medical interventions, surgical procedures, and other newer forms of treatment such as stem cell therapy, which suggests a differing pathogenic basis to PFCD^10^. Inadequate surgical procedures may result in significant morbidity such as a risk of fecal incontinence^11^. Given that these two types of fistulas significantly different management in pathogenesis, complexity, and treatment, therefore, it is of crucial importance to differentiate between these two types of fistulas. Currently, endoscopy and magnetic resonance imaging (MRI) represent the conventional diagnostic gold standards for differentiating PFCD from CAF. Nevertheless, their inherent invasiveness and substantial cost often restrict widespread clinical utilization^12^. Given the advantage of noninvasiveness, there is an urgent need to identify novel highly efficient and specific biomarkers to improve the non-invasive diagnostic accuracy of perianal fistulas.

Emerging evidence indicates that dysregulation of metabolic pathways—particularly amino acid metabolism—is mechanistically linked to the pathogenesis of Crohn’s disease (CD)^13^. Nutritional deficiencies exhibit notably high frequency in Crohn’s disease (CD) populations (approximating 65–75%) ^14^. Amino acids, fundamental nitrogenous compounds composed of carbon, hydrogen, oxygen, nitrogen, and sulfur, execute indispensable physiological roles spanning protein synthesis, energy homeostasis, immune regulation, and neurotransmitter synthesis^15^. Amino acids are indispensable for intestinal development and mucosal integrity preservation, critically supporting barrier function^16^. Intestinal mucosal cells utilize these compounds as essential precursors for synthesizing metabolically active proteins, glutathione (GSH), nitric oxide, polyamines, and related biomolecules. Furthermore, they serve as substrates for macromolecular biosynthesis during mucosal repair and provide metabolic energy for enterocytes^17^. Gut microbiota metabolize amino acids to generate proteins and diverse bioactive metabolites, constituting vital mechanisms for maintaining intestinal homeostasis and attenuating inflammatory responses. ^18^. Emerging evidence suggests that specific amino acids, notably glutamine and arginine, may modulate Crohn’s disease (CD) progression. These compounds appear to exert anti-inflammatory and antioxidative effects, potentially suppressing proinflammatory cytokine production^19^.

Metabolic disturbance is one of the main characteristics of PFCD patients^20,21^. Some abnormal metabolites in blood may be biomarkers for the induction and development of PFCD. Given the close association between amino acid metabolism and the pathogenesis of perianal fistula, an in-depth investigation into the amino acid metabolic profiles of newly diagnosed and treatment-naïve PFCD and CAF patients will not only help to elucidate the pathophysiological nature of these two diseases but may also identify potential diagnostic biomarkers. The present study employed LC-MS/MS to quantitatively analyze 25 serum amino acids in patients with PFCD and CAF. Through multivariate statistical analyses (including PCA and PLS-DA), we systematically investigated the remodeling characteristics of amino acid metabolic networks during fistula pathogenesis, aiming to identify the critical nodes driving their distinct etiological mechanisms. Furthermore, this study is dedicated to identifying serum amino acid biomarkers that can effectively differentiate between PFCD and CAF. The aim is to provide clinical diagnostics with molecular tools characterized by high specificity and sensitivity. This research not only contributes to the optimization of treatment strategy formulation, enabling precision medicine, but also offers novel targets and directions for subsequent targeted interventions.

## Materials and methods

### Patient selection

A total of 36 patients with new-onset PFCD and 36 gender- and age-matched patients with CAF were retrospectively enrolled from the Department of Proctology at Longhua Hospital, Shanghai University of Traditional Chinese Medicine, between June 2023 and May 2024. The identification of PFCD adhered to recognized consensus standards, relying on integrated assessment of clinical parameters, radiological appearances, laboratory markers, endoscopic characteristics, and histological examination^22^. This was a cross-sectional observational study, which included newly diagnosed and treatment-native patients with PFCD and CAF. Demographic and clinical characteristics were recorded for all participants, including age, sex, weight, BMI, smoking status, comorbidities, age at disease onset, disease location, disease duration, medication history, prior/current therapies, fistula characteristics (number), and endoscopic/radiographic assessment data. Serum and fecal inflammatory biomarkers—specifically fecal calprotectin (FC), C-reactive protein (CRP), and erythrocyte sedimentation rate (ESR)—were quantitatively assessed.

Ethical approval for this study was obtained from the Institutional Review Board of Longhua Hospital, Shanghai University of Traditional Chinese Medicine (Shanghai, China; Approval no.: 2020LCSY032), in accordance with the Declaration of Helsinki principles. All blood samples were collected in the morning between 7:00–9:00 am after an overnight fast (at least 8 h). Venous blood samples were drawn into clot activator tubes (Somerset, NJ, USA), allowed to coagulate at ambient temperature for 30 min, and centrifuged at 3,000 ×g for 10 min. Harvested serum aliquots were cryopreserved at -80 °C pending analysis.

### Inclusion and exclusion criteria

Participants with PFCD met the following inclusion criteria: (a) definitive PFCD diagnosis confirmed through clinical assessment combined with endoscopic, histopathologic, MRI, and/or biochemical biomarkers; (b) ≥ 1 actively discharging perianal fistula tract. Exclusion criteria comprised: (a) anal strictures or abscesses requiring urgent surgical intervention, (b) malignancy-, trauma-, or human immunodeficiency virus (HIV)-associated secondary fistulas, and (c) concurrent infectious gastroenteritis. (d) severe liver or kidney dysfunction. (e) History of recent major trauma or surgery (within the past 3 months), which may affect the body’s metabolic state. Inclusion criteria for CAF cases required: (a) ≥ 1 perianal fistula with active purulent discharge; (b) negative inflammatory bowel disease status verified by colonoscopy, pelvic MRI. Exclusion criteria comprised: (a) pilonidal disease involving sacrococcygeal region, (b) fistulae secondary to malignancy, traumatic injury or human HIV infection, (c) active infectious gastroenteritis, and (d) History of recent major trauma or surgery (within the past 3 months), which may affect the body’s metabolic state. (e) severe liver or kidney dysfunction.

### Clinical evaluations

To evaluate disease severity, the Perianal Disease Activity Index (PDAI) and Crohn’s Disease Activity Index (CDAI) were employed. The CDAI total score ranges from 0 to 600, with higher scores correlating with increased severity: remission (< 150), mild (150–220), moderate (221–450), and severe (> 450)^23^. The PDAI, considered the most reliable tool for assessing perianal disease severity, combines patient-reported symptoms with clinical examination findings. It evaluates five components: discharge, pain/activity limitation, sexual activity restriction, perianal lesion type, and induration severity, with total scores ranging from 0 to 20. A PDAI score ≤ 4 suggests inactive disease, whereas > 4 signifies active fistulizing disease^24^.

### Chemicals and reagents

Twenty-five amino acid standards, including 2-aminoisobutyric acid (AIBA), alanine (Ala), arginine (Arg), asparagine (Asn), aspartic acid (Asp), citrulline (Cit), cysteine (Cys), glutamine (Gln), glutamic acid (Glu), glycine (Gly), histidine (His), trans-4-hydroxyproline (hPro), leucine (Leu), isoleucine (Ile), lysine (Lys), methionine (Met), ornithine (Orn), phenylalanine (Phe), proline (Pro), sarcosine (Sar), serine (Ser), threonine (Thr), tryptophan (Trp), tyrosine (Tyr), and valine (Val), were purchased from Bide Pharmatech Ltd. (Shanghai, China). Five internal standards (IS), including Ala-D4, Glu-D5, Phe-D5, Ser-D3, and Trp-D5 were obtained from Cambridge Isotope Laboratories, Inc. (Andover, MA, USA). HPLC-grade acetonitrile and formic acid were acquired from Merck KGaA (Darmstadt, Germany). Ultrapure water was produced utilizing a Millipore purification apparatus (Merck Millipore, MA, USA).

### Preparation of calibration and quality control samples

The analyte stock solution containing 25 target amino acids and the internal standard (IS) stock solution containing five isotopically labeled amino acids were prepared in 0.1 M HCl. In the analyte stock solution, the concentrations of Cys, His, and Lys were 2 mg/mL, while the remaining 22 amino acids were each at a concentration of 0.4 mg/mL. Each isotopic internal standard in the IS stock solution was prepared at 1 mg/mL. Subsequently, the analyte stock solution was serially diluted with purified water to generate the working solutions for the preparation of calibration standards and quality control (QC) samples. The IS stock solution was diluted with acetonitrile: water (v/v, 4: 1) to obtain an IS working solution at a concentration of 100 ng/mL. Then, 150 µL of simulated serum, prepared according to a previously reported method^25,26^, was spiked with 50.0 µL of the corresponding working solution to yield the final calibration standards and QC samples. For Cys, His, and Lys, the calibration standards were prepared at concentrations of 0.5 (lower limit of quantification, LLOQ), 1.0, 2.5, 10, 50, 200, 400, and 500 µg/mL (upper limit of quantification, ULOQ), with QC samples at 1.5 (low QC, LQC), 30 (medium QC, MQC), and 300 µg/mL (high QC, HQC). For the other 22 amino acids, the calibration standards were 0.1 (LLOQ), 0.2, 0.5, 2.0, 10, 40, 80, and 100 µg/mL (ULOQ), with QC samples at 0.3 (LQC), 6 (MQC), and 60 µg/mL (HQC).

### Sample pretreatment

A 5 µL aliquot of the calibration standard, QC, or test sample was mixed with 95 µL of the IS working solution. The mixture was vortexed for 5 min and then centrifuged at 12,000 rpm for 10 min at 4 °C. Subsequently, 50 µL supernatant was transferred, combined with 50 µL water, and vortex-mixed thoroughly. Finally, 1 µL of the reconstituted solution was injected into the LC-MS/MS system for analysis.

### Method validation

The LC-MS/MS method for quantifying 25 amino acids in human serum was validated in compliance with the bioanalytical method validation guidelines issued by the U.S. FDA and EMA^27,28^. The validation parameters covered linearity, intra-day and inter-day precision and accuracy, all evaluated at the lower limit of quantification (LLOQ) and three quality control concentration levels (LQC, MQC, HQC). Additionally, extraction recovery, matrix effect, and stability were systematically assessed at each of the aforementioned QC concentrations.

### Sample analysis

Sample analysis was conducted using an LC-MS/MS system comprising a LC-20 A liquid chromatograph (Shimadzu, Kyoto, Japan) and a Triple Quad 4500 mass spectrometer (Applied Biosystems Sciex, Massachusetts, USA). Chromatographic separation was performed on a Waters XBridge HILIC column (100 mm × 4.6 mm, 3.5 μm) with a mobile phase consisting of (A) water with 0.2% formic acid and (B) acetonitrile with 0.1% formic acid. The elution program was as follows: 0–2 min, gradient from 65% to 35% B; 2–3 min, maintained at 35% B; 3.0–3.1 min, gradient from 35% to 60% B; 3.1–4.5 min, maintained at 60% B. The column temperature was maintained at 40 °C, and the flow rate was set to 0.8 mL/min. Mass spectrometry conditions were optimized as follows: ion source was positive ion electrospray ionization (ESI+); detection mode was multiple reaction monitoring (MRM); ion source temperature was 550 °C; ion spray voltage was 5500 V; curtain gas (CUR) pressure was 40 psi; nebulizer gas (Gas 1) pressure was 55 psi; auxiliary heater gas (Gas 2) pressure was 60 psi. Additional specific mass spectrometry parameters are detailed in Table 1. Furthermore, to prevent systematic bias from the analytical sequence, all samples were randomized and analyzed with an inter-dispersed QC strategy, whereby LQC, MQC, and HQC samples were injected after every 9 clinical samples, establishing a closed-loop quality control system to ensure data accuracy.

**Table 1:** The mass spectrometry parameters of amino acids.

Amino acid	MRM Transition	Collision Energy (eV)	Decluster Potential (V)	Dwell time (msc)
AIBA	104.0 > 57.9	15	20	20
Ala	89.9 > 44.1	19	35	20
Arg	175.0 > 116.1	19	30	20
Asn	133.0 > 73.8	22	30	20
Asp	134.1 > 74.0	19	20	20
Cit	176.0 > 159.1	14	20	20
Cys	122.1 > 59.0	37	33	20
GIn	147.2 > 130.1	14	17	20
Glu	148.0 > 84.0	22	49	20
Gly	76.0 > 30.0	17	28	20
His	155.9 > 109.8	25	30	20
hPro	132.1 > 85.8	18	20	20
Leu/Ile	132.0 > 86.0	14	37	20
Lys	147.3 > 130.0	16	20	20
Met	150.0 > 133.0	13	40	20
Orn	133.0 > 70.0	22	20	20
Phe	166.0 > 119.7	22	48	20
Pro	116.0 > 69.8	24	47	20
Sar	90.3 > 44.0	13	10	20
Ser	106.0 > 60.0	16	19	20
Thr	120.0 > 74.0	13	28	20
Trp	205.0 > 188.2	11	58	20
Tyr	182.0 > 136.1	18	40	20
Val	118.0 > 72.0	15	51	20
Ala-D4 (IS)	94.0 > 48.0	15	33	20
Glu-D5 (IS)	153.1 > 89.1	22	20	20
Phe-D5 (IS)	170.9 > 125.0	27	70	20
Ser-D3 (IS)	109.1 > 63.0	16	30	20
Trp-D5 (IS)	210.0 > 192.1	14	30	20

### Data processing

LC-MS/MS data were initially processed using Analyst 1.6.3 software. The concentrations of amino acids in serum samples were determined using calibration curves constructed via the internal standard method, applying 1/*x*² weighting factor. Statistical analyses were carried out with SPSS 25.0 (IBM Corp., Chicago, IL, USA). The Shapiro-Wilk test was employed to evaluate the data distribution. Measurement data with normal distribution were expressed as mean ± standard deviation (M ± SD) and analyzed between groups using the t-test. Non-normally distributed variables were presented as median and interquartile range (IQR) and the Mann - Whitney rank -sum test was applied for comparison. Multivariate statistical analysis and visualization of the serum amino acid data were carried out using SIMCA-P 14.1 software. Prior to conducting PCA and OPLS-DA analyses, all quantitative data were subjected to normalization using isotopically labeled internal standards followed by Pareto scaling preprocessing (where each variable is divided by the square root of its standard deviation), aiming to reduce the impact of concentration heterogeneity while preserving the data structure.

## Results

### Patient characteristics

**Fig. 1:**
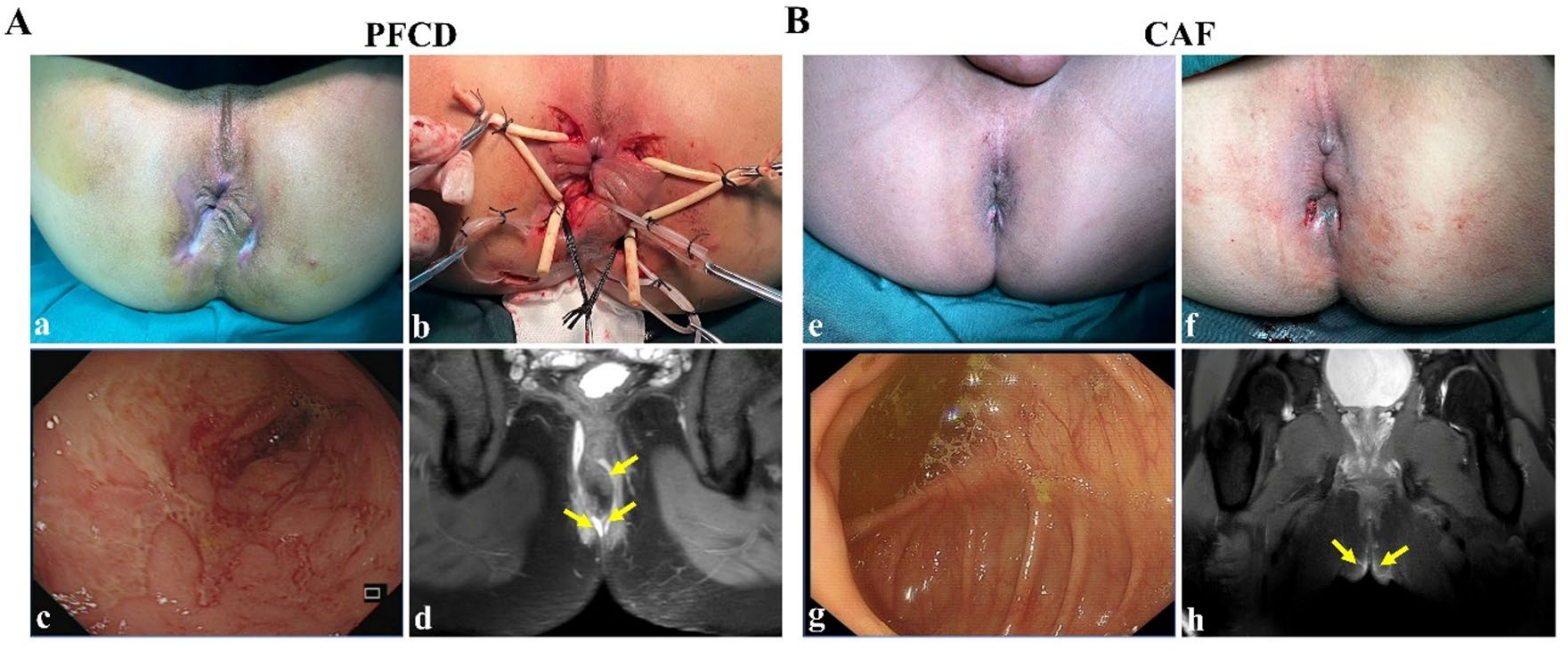
Clinical Characteristics of PFCD (**A**) and CAF (**B**). a, e: Pre-treatment of PFCD and CAF; b, f: Post-treatment of PFCD and CAF; c, g: Representative endoscopic picture captures of colon; d, h: perianal MRI (coronal T2WI-fat suppression), with fistula tracts (yellow arrow).

Baseline characteristics demonstrated no statistically significant differences between the PFCD and CAF cohorts regarding gender distribution, age, body weight, fistula location, BMI, smoking history, medication, comorbidities or symptom duration (all *p* > 0.05). Applying the Montreal classification system to 36 PFCD patients revealed the following distributions: Age stratification: Group A1 (< 17 years): 5 cases (13.9%); A2 (17–40 years): 26 cases (72.2%); A3 (> 40 years): 5 cases (13.9%). Disease localization: L1 (isolated ileal): 8 patients (22.2%); L2 (colonic only): 8 (22.2%); L3 (ileocolonic): 20 (55.6%). Behavioral phenotypes: B1 (inflammatory/nonstricturing): 10 patients (27.8%); B2 (stricturing): 26 (72.2%); no B3 (penetrating) cases observed. Compared to CAF controls, PFCD patients demonstrated significantly elevated levels of inflammatory biomarkers: FC, CRP, and ESR (*p* < 0.001). Complete demographic and clinical profiles are detailed in Fig. 1; Table 2.

**Table 2:** Demographic and clinical parameters.

Variable	PFCD(*n* = 36)	CAF(*n* = 36)	*p*
Age (years)	28.47 ± 1.50	29.50 ± 1.38	>0.05
Sex (Male/Female)	29/7	28/8	>0.05
Weight (kg) BMI Smoking history Number of fistulas (IQR) Age at diagnosis	64.17 ± 1.22 20.17 ± 2.79 0 (0.00) 2.0 (2.00–3.00)	62.72 ± 1.56 20.81 ± 1.96 0 (0.00) 1.0 (1.00–2.00) N/A	>0.05 >0.05 >0.05
A1 (<17 years)	5 (13.9%)	3 (8.3%)	
A2 (17–40 years)	26(72.2%)	28 (78.1%)	
A3 (>40 years) Disease location	5(13.9%)	5 (13.6%) N/A	
L1 (ileum)	8 (22.2%)	-	
L2 (colon)	8 (22.2%)	-	
L3 (ileum-colon) L4 (upper gastrointestinal) Disease behavior	20 (55.6%) 0	- - N/A	
B1 (nonstenotic, nonpenetrating)	10 (27.8%)		
B2 (stenotic) B3 (penetrating) p (perianal) CDAI median (IQR) PDAI median (IQR) FC median (IQR) CRP median (IQR) ESR median (IQR) Medication Steroids Thiopurine Biologics Antibiotics Comorbidities Diabetes Liver or renal dysfunction	26 (72.2%) 0 36(100%) 179.0 (132.00-261.75) 10.44 ± 3.13 180.0(113.00-285.75) 8.45 (4.44–13.56) 37.07 ± 19.70 0 0 0 5 (13.9%) 0	36(100%) N/A N/A 32.5(25.50–55.2) 2.00(0.90–3.48) 14.97 ± 8.15 6 (16.7%) 0	<0.001 <0.001 <0.001

## Method validation

The calibration curves for the 25 target amino acids were constructed using isotopically labeled internal standards, as detailed in Table S1. All curves demonstrated a correlation coefficient (R^2^) greater than 0.99. As shown in Table S2, the intra-day and inter-day accuracy for all amino acids across the four concentration levels ranged from 86.9% to 110.1% and 91.9% to 105.0%, respectively, with the corresponding precision (expressed as R.S.D.) being below 14.0% and 11.9%. The results for extraction recovery and matrix effect, summarized in Table S3, showed that at the three QC levels, the recoveries were between 92.1% and 109.9% (R.S.D. < 10.5%), and the matrix effects were between 89.3% and 107.2% (R.S.D. < 12.6%). Stability assessment results (Table S4) indicated that serum samples at three QC concentrations remained stable under various conditions: after being kept at room temperature for 3 h (accuracy: 94.9% to 106.4%; R.S.D. < 11.0%) and after undergoing three freeze-thaw cycles (accuracy: 93.1% to 108.4%; R.S.D. < 11.9%). In conclusion, all validation results met the requirements of bioanalytical guidance, adequately demonstrating that the method possesses good accuracy and precision, high extraction recovery, acceptable matrix effect, and excellent sample stability for the quantification of human serum samples.

### Serum amino acid quantification

Serum amino acid profiling revealed significantly higher total amino acid concentrations in the CAF cohort compared to the PFCD group (*p* < 0.05). Differential regulation was observed in 13 specific amino acids between the two groups. Specifically, compared to the CAF group, only Cys concentration was significantly elevated in the PFCD group, while twelve amino acids, including AIBA, Ala, Asn, Asp, Glu, His, Leu, Orn, Phe, Sar, Tyr, and Val, demonstrated significantly lower concentrations in the PFCD group relative to the CAF group (Table 3). These findings suggest that the amino acid metabolic profile may serve as potential biomarkers for differentiating between CAF and PFCD.

**Table 3:** The serum amino acid concentrations in the PFCD and CAF groups (x ± SD, *n* = 36).

Analyte	PFCD (µg/mL)	CAF (µg/mL)	*P*	Fold Change^*^
AIBA^#^	1.53 ± 0.46	2.02 ± 0.39	0.0003	1.320
Ala^#^	36.8 ± 8.52	42.22 ± 7.95	0.0283	1.147
Arg	29.47 ± 6.85	25.5 ± 6.79	0.0588	0.865
Asn^#^	7.41 ± 1.56	8.43 ± 1.62	0.0261	1.138
Asp^#^	8.13 ± 3.55	11.98 ± 3.16	0.0003	1.474
Cit	4.23 ± 0.85	4.23 ± 0.92	0.9972	1.000
Cys^#^	29.53 ± 5.93	21.76 ± 4.22	< 0.0001	0.737
GABA	0.39 ± 0.2	0.3 ± 0.12	0.0739	0.769
Gln	68.68 ± 10.62	64.5 ± 6.56	0.1028	0.939
Glu^#^	16.21 ± 7.65	30.85 ± 9.59	< 0.0001	1.903
Gly	20.72 ± 4.09	20.38 ± 3.83	0.7801	0.984
His^#^	22.69 ± 5.08	30.91 ± 4.94	< 0.0001	1.362
hPro	2.87 ± 1.27	2.71 ± 1.17	0.6790	0.944
Ile/Leu^#^	34.8 ± 7.79	43.23 ± 7.03	0.0001	1.242
Lys	27.63 ± 4.26	28.5 ± 4.21	0.4747	1.031
Met	4.33 ± 0.85	4.71 ± 0.82	0.1239	1.088
Orn^#^	22.46 ± 7.79	36.46 ± 9.51	< 0.0001	1.623
Phe^#^	12.21 ± 2.99	14.73 ± 2.76	0.0063	1.206
Pro	30.87 ± 8.79	26.44 ± 5.53	0.0512	0.856
Sar^#^	28.91 ± 5.97	33.56 ± 5.74	0.0097	1.161
Ser	17.68 ± 3.93	19.22 ± 2.23	0.0881	1.087
Thr	16.06 ± 3.16	16.19 ± 2.59	0.8847	1.008
Trp	10.87 ± 2.13	10.79 ± 1.27	0.8754	0.993
Tyr^#^	13.84 ± 2.92	17.25 ± 2.75	0.0002	1.246
Val^#^	34.39 ± 6.33	43.31 ± 5.31	< 0.0001	1.259

Data are presented as mean ± standard deviation (SD). Group comparisons were performed using Student’s *t*-test. ^#^denotes amino acids with significant differences between the PFCD and CAF groups. Fold Change represents the ratio of the mean concentration in the CAF group to that in the PFCD group.

### Principal component analysis

**Fig. 2:**
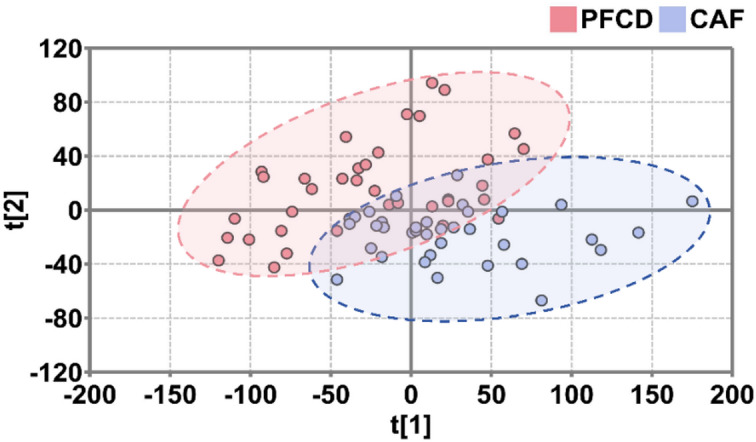
PCA score plot of serum amino acid profiles in rats from PFCD and CAF groups (*n* = 36).

Principal component analysis (PCA) was performed on a total of 72 samples from the PFCD and CAF groups, using the concentrations of various amino acids as variables. The first two principal components (PC1 and PC2) cumulatively explained 77.1% of the variance in the original data, indicating that the model had good explanatory power. As shown in Fig. 2, the distribution of sample points in the PCA plot exhibited partial overlap between the PFCD and CAF groups, but a clear separation trend was observed overall: the sample points of the PFCD group were predominantly concentrated in the left region, while those of the CAF group were mainly located in the right region. This separation trend suggests significant differences in the amino acid metabolic profiles between the PFCD and CAF groups, indicating distinct metabolic patterns of amino acids in the two groups. These results further support the potential of amino acids as biomarkers for distinguishing between PFCD and CAF.

### Orthogonal partial least squares-discrimination analysis

**Fig. 3:**
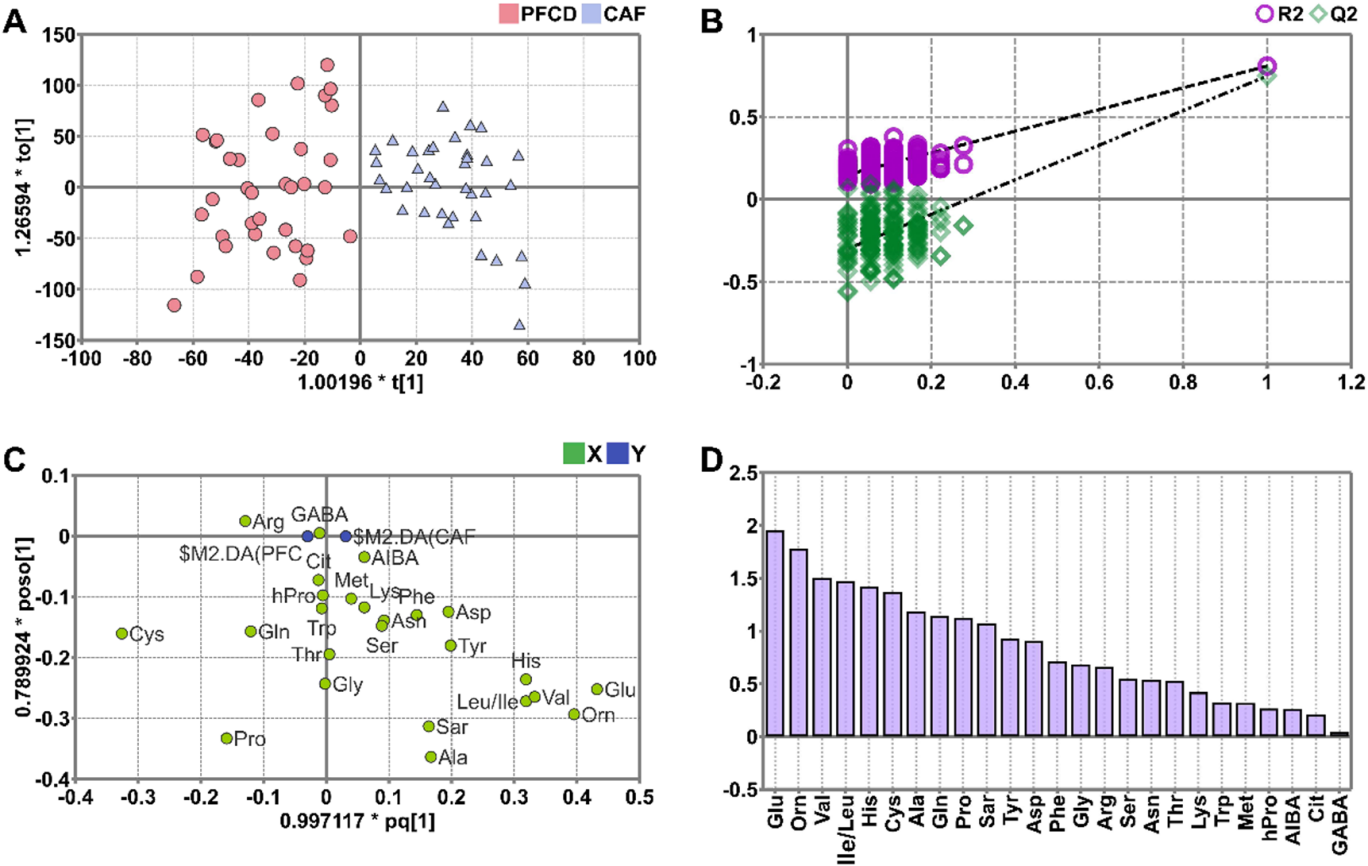
The OPLS-DA model of serum amino acids between PFCD and CAF groups. (**a**) Score plot; (**b**) Permutation test plot; (**c**) Loading plot; (**d**) VIP plot.

OPLS-DA was performed on 72 samples from the PFCD and CAF groups using amino acid concentrations as the X-variable and group classification as the Y-variable, with two principal components. As shown in Fig. 3a, the OPLS-DA score plot revealed a clear separation trend between the PFCD grup (red markers) and CAF group (blue markers) in metabolic profiles, further confirming distinct amino acid metabolic patterns between the two groups. Model quality was evaluated using three key parameters: R²X (interpretation of X-variation), R²Y (interpretation of Y-discrimination), and Q² (predictive accuracy). Generally, models are considered robust when R²Y and Q² exceed 50% ^29^. In this study, the OPLS-DA model parameters were R²X = 76.7%, R²Y = 80.8%, and Q² = 74.8%, indicating strong explanatory power for both X and Y variables and high predictive accuracy. To validate model reliability and avoid overfitting, a 200-iteration permutation test was conducted. The permuted R²Y and Q² values were lower than the original values, and the Q² regression line intercept was negative (Fig. 3b), confirming the absence of overfitting and demonstrating the model’s stability and predictive capability^30^. Potential biomarkers associated with metabolic differences were screened using the OPLS-DA loading plot (Fig. 3c) and Variable Importance in Projection (VIP, Fig. 3d). The loading plot highlighted amino acid variables contributing to group discrimination, with variables distant from the origin representing key contributors. VIP scores quantified variable importance, with VIP > 1.0 considered significant. Ten amino acids (Ala, Cys, Gln, Glu, His, Leu, Orn, Pro, Sar, and Val) exhibited VIP scores > 1.0. Subsequent t-tests identified significant differences (*p* < 0.05) in eight amino acids (excluding Gln and Pro) between PFCD and CAF groups (Table 2), suggesting these eight amino acids as potential biomarkers for distinguishing the two groups.

### Metabolic pathway enrichment analysis

**Fig. 4:**
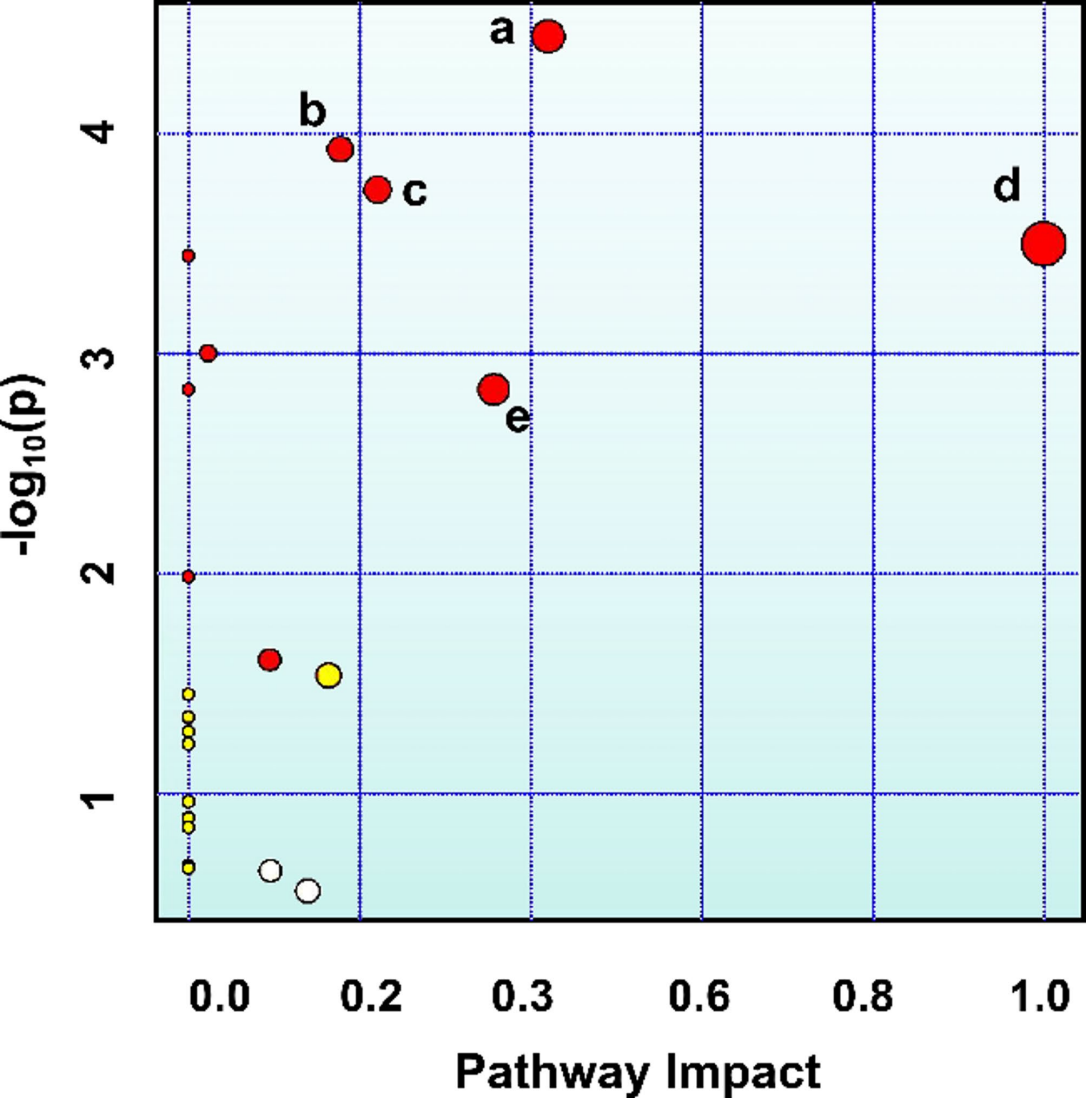
KEGG Enrichment pathways of differential serum amino acids between PFCD and CAF groups. (**a**) Alanine, aspartate and glutamate metabolism; (**b**) Arginine biosynthesis; (**c**) Histidine metabolism; (**d**) Phenylalanine, tyrosine and tryptophan biosynthesis; (**e**) Phenylalanine metabolism.

To investigate the pathogenic mechanism differences between the CAF and PFCD groups from the perspective of amino acid metabolism, this study utilized the differential amino acid data extracted from Table 2 and performed metabolic pathway enrichment analysis via the MetPA metabolomics platform (http://www.metaboanalyst.ca/) based on the KEGG database. Metabolic pathways with a pathway impact value > 0.1 and a significance threshold of -log10(*P*) > 2 (corresponding to *P* < 0.01) were selected as key pathways. The results demonstrated that five pathways-alanine, aspartate, and glutamate metabolism; arginine biosynthesis; histidine metabolism; phenylalanine, tyrosine, and tryptophan biosynthesis; and phenylalanine metabolism—showed significant associations with metabolic disparities between the two groups (Fig. 4). These pathways may act as critical metabolic determinants underlying the pathogenic divergence between PFCD and CAF by regulating imbalances in key amino acid metabolic networks, thereby offering novel insights into their pathophysiological distinctions.

## Discussion

In this study, quantitative analysis was performed on 25 serum amino acids from patients with CAF and PFCD. The serum amino acid metabolic profiles of the two groups were compared to investigate differences in their pathogenesis and to identify potential diagnostic biomarkers. The results demonstrated a distinct divergence in serum amino acid metabolic profiles between PFCD and CAF patients. These findings not only provide a novel molecular perspective for differentiating between these two diseases—which share similar clinical manifestations but have distinct pathological bases—but also offer valuable insights for further elucidating their respective pathogenic mechanisms.

This study demonstrated that, when compared with CAF patients, PFCD patients exhibited lower overall serum amino acid concentrations. Specifically, significant differences were detected in the levels of 13 amino acids, namely AIBA, Ala, Asn, Asp, Cys, Glu, His, Leu, Orn, Phe, Sar, Tyr, and Val. Notably, while Cys was significantly elevated in PFCD patients, the remaining 12 amino acids were present at significantly lower concentrations in their serum compared to CAF patients. This overall pattern is closely consistent with the pathophysiological characteristics of PFCD. As an extra-intestinal manifestation of CD, PFCD stems from systemic immune dysregulation, which is characterized by the hyperactivation of the Th1/Th17 axis and chronic intestinal inflammation^31^. This persistent systemic inflammation propels the body into a hypermetabolic and catabolic state, which in turn requires a substantial consumption of amino acids for three main purposes. Firstly, amino acids are needed for the proliferation and maintenance of the function of immune cells^32,33^. Secondly, they are essential for the synthesis of acute - phase proteins^34^. Thirdly, they play a crucial role in the repair of damaged intestinal and perianal tissues^35^. Moreover, CD patients frequently encounter nutrient malabsorption, which further exacerbates the amino acid deficiency^36,37^. Therefore, the widespread reduction in serum amino acid levels among PFCD patients likely reflects the combined effects of systemic inflammatory burden, increased catabolism, and underlying nutrient absorption impairment. In contrast, CAF is triggered by localized inflammation resulting from anal gland infection^38^. Although an inflammatory response is also involved, the scope of this inflammation is relatively limited, with minimal systemic impact. This can well explain why CAF patients generally have higher serum amino acid levels than PFCD patients. The elevated amino acid levels in CAF patients may suggest a relatively normal systemic metabolic state or the release of amino acids from locally damaged tissues into the circulation.

The phenomenon that Cys is significantly elevated in PFCD while reduced in CAF is worthy of particular attention. Mechanistically, the significant elevation of cysteine in PFCD patients may be associated with an adaptive response within the glutathione redox cycle. Specifically, Cys acts as the rate - limiting precursor for glutathione (GSH) synthesis^39^. Its accumulation might be due to two possible reasons. On one hand, it could be a compensatory up - regulation in response to sustained oxidative stress. On the other hand, it may indicate an impairment in GSH synthesis or recycling. As previously demonstrated by studies, chronic inflammation in IBD is closely associated with glutathione depletion and altered redox homeostasis^40^. In this context, the elevated cysteine may represent a compensatory mechanism that fails to maintain GSH levels. Consequently, this failure contributes to persistent oxidative tissue injury and impaired fistula healing in PFCD. Furthermore, it is plausible that the observed systemic amino acid alterations partly originate from or are modulated by shifts in gut microbial metabolic activity. As highlighted by Adegbola, the gut microbiome plays a critical role in host amino acid metabolism, and dysbiosis in Crohn’s disease can significantly influence microbial production or consumption of key amino acids, including tryptophan, branched-chain amino acids, and those involved in sulfur metabolism. The distinct metabolic signature in PFCD may thus reflect a complex interplay between host inflammatory responses and microbiome-derived metabolic perturbations.

Amino acids related to energy and nitrogen metabolism, namely Ala, Asp, and Glu, are present at low levels in PFCD. This finding is consistent with the enrichment of the “Alanine, aspartate, and glutamate metabolism” pathway. This pathway is involved in the tricarboxylic acid cycle and nitrogen transport^41^. Given the high - metabolic state of PFCD and the increased energy demand, it is reasonable to infer that a significant depletion of these amino acids occurs, as they are utilized for energy production or the synthesis of other essential molecules. Branched - chain amino acids (BCAAs), including Leu and Val, are reduced in PFCD. BCAAs are mainly metabolized in muscle, and their decreased levels are often regarded as an indicator of increased muscle catabolism for energy and protein synthesis^42^. This observation aligns with the muscle wasting and premalignant state commonly seen in CD patients. Both aromatic amino acids (Phe, Tyr) and His are at low levels in PFCD, and the associated pathways (Phenylalanine, tyrosine, and tryptophan biosynthesis; Phenylalanine metabolism; Histidine metabolism) are significantly enriched. These amino acids serve as precursors of neurotransmitters, hormones (e.g., catecholamines), and inflammatory mediators (e.g., histamine)^43,44^. Their depletion may be associated with the dysregulation of the neuro - immune - endocrine network in PFCD. Moreover, inflammation may heighten the need for their synthesis^45^. In particular, although tryptophan (Trp) does not show a significant difference in this study, its metabolism (e.g., the kynurenine pathway) has been demonstrated to be abnormally active in CD and involved in immune regulation. Changes in the levels of Phe/Tyr, as part of the relevant pathways, indirectly reflect this metabolic remodeling^46,47^. Arg is a precursor for NO synthesis, which plays a dual role in immunomodulation and inflammation^48,49^. Although the difference in Arg itself is not significant, the decrease in its metabolite Orn may imply an alteration in Arg metabolic flow (e.g., NO synthesis vs. urea cycling) in the chronic inflammatory setting of PFCD. This alteration may, in turn, affect vascular function, immune cell activity, and tissue repair^50^. Sar levels are low in PFCD. Sar is associated with one - carbon unit metabolism (folate cycle, methionine cycle)^51^. The changes in its levels may reflect disturbances in PFCD related to basic metabolic processes such as methylation and nucleotide synthesis, which could be influenced by chronic inflammation or intestinal dysbiosis.

Both PCA and OPLS-DA analyses demonstrated a clear separation trend in amino acid profiles between PFCD and CAF groups, indicating the potential of these profiles to differentiate between the two diseases. The OPLS-DA model exhibited excellent goodness-of-fit (R²X, R²Y) and predictive ability (Q²), and passed the permutation test, confirming its stability and reliability. Through VIP value screening and t-test validation, a panel of eight amino acids, namely Ala, Cys, Glu, His, Leu, Orn, Sar, and Val, was identified as potential biomarkers for distinguishing PFCD from CAF. Compared with single markers, this multi-metabolite combination is likely to offer higher diagnostic accuracy and robustness. This finding holds significant clinical implications, as current diagnostic methods relying on clinical assessment, imaging, and pathology have limitations, particularly in early-stage or atypical cases. Developing a minimally invasive diagnostic tool based on serum amino acid profiles could enable early and precise differentiation between PFCD and CAF, thereby guiding subsequent treatment strategies (e.g., PFCD requiring immunosuppressants or biologics, while CAF primarily necessitating surgical intervention) and reducing misdiagnosis.

The five key metabolic pathways identified through enrichment analysis, namely alanine, aspartate, and glutamate metabolism; arginine biosynthesis; histidine metabolism; phenylalanine, tyrosine, and tryptophan biosynthesis; and phenylalanine metabolism, all converge on amino acid networks intricately linked to energy metabolism, nitrogen homeostasis, inflammatory responses, oxidative stress, and immune regulation. Alterations in these pathways are not merely consequences of disease states but may also reciprocally drive disease progression. For instance, energy metabolism dysregulation could exacerbate tissue damage and impede repair processes. Imbalances in Arg/Orn metabolism might compromise immune cell function and microvascular integrity. Changes in Phe/Tyr/Trp/His metabolism could modulate the gut and perianal immune microenvironment via neurotransmitters, hormones, or immunomodulatory metabolites such as kynurenine. These findings provide a molecular-level explanation for how systemic immune dysregulation in PFCD impacts amino acid metabolism and how localized infection in CAF elicits distinct metabolic perturbations, thus highlighting potential targets for future research and intervention.

Amino acid measurements can serve as a valuable adjunct to existing diagnostic approaches. Specifically, endoscopy, being an invasive procedure, may not be well-tolerated by all patients, particularly those suffering from severe CD. Moreover, MRI is not only expensive but also not readily accessible in all healthcare settings. Although FC testing is non-invasive, it has limitations in terms of specificity. In contrast, amino acid measurements, which are non-invasive, can be effectively employed as a preliminary screening tool. If the amino acid profile suggests a high probability of PFCD or CAF, then further invasive or costly tests like endoscopy or MRI can be selectively carried out. This approach can significantly reduce the burden on both patients and healthcare resources. Furthermore, by integrating amino acid measurements with endoscopy, MRI, or fecal calprotectin testing, we can enhance the accuracy of diagnosis. In fact, in certain situations, amino acid measurements may potentially substitute for some existing diagnostic tests. For instance, for patients who cannot undergo endoscopy or MRI due to contraindications, amino acid testing emerges as a viable alternative. In terms of clinical decision-making, for example, when the amino acid levels fall within the normal range, it indicates that the probability of a complex fistula is likely to be low. In such cases, conservative management can be reasonably considered. On the contrary, if the amino acid levels are significantly abnormal, then it becomes necessary to initiate further diagnostic tests or more aggressive treatment strategies.

In this study, LC - MS/MS, a technique renowned for its high sensitivity and specificity, was employed to accurately quantify multiple amino acids. By integrating multivariate statistical methods and pathway analysis, robust support was furnished for the study results. However, it is important to acknowledge that this study has several limitations. Firstly, all the perianal fistulas patients in our study were recruited from a single center. It is noteworthy that the limited sample size can potentially hinder the generalizability of the identified biomarkers to a wider population. Meanwhile, we must also take into account that the amino - acid metabolic profiles we observed are likely to be affected by a multitude of factors, such as genetic predispositions, environmental exposures, and dietary habits. The cross - sectional design of the study fails to uncover causal relationships or dynamic changes. Additionally, the analysis was confined to serum, overlooking the metabolic characteristics of local tissues such as fistulas and intestinal mucosa. Furthermore, the diagnostic efficacy of the screened potential biomarkers remains to be verified. Therefore, future research endeavors should focus on expanding the sample size and diversifying the biosamples, including blood, urine, feces, and tissue. Conducting multicenter and longitudinal studies is essential to track the dynamic changes in amino acid profiles, and determine whether these changes are associated with disease progression, remission, or treatment response. Integration of tissue/space metabolomics and gut metagenomics, along with functional experiments, is required to confirm the roles of key amino acids. Ultimately, the clinical application value of the biomarker combination should be thoroughly evaluated.

## Conclusion

In summary, this study harnessed LC - MS/MS technology in conjunction with multivariate statistical analyses to clearly distinguish the distinct serum amino acid profiles between patients suffering from PFCD and those with CAF. Based on our findings, the distinct metabolic profiles of amino acids could be incorporated into clinical practice as a preliminary screening tool. For instance, a plasma-based metabolic panel could be utilized in patients presenting with perianal symptoms prior to advanced imaging such as MRI. This approach may help distinguish between pfCD and CAF at an earlier stage, potentially guiding subsequent diagnostic and therapeutic decisions. In suspected cases, abnormal amino acid profiles could prompt earlier referral for MRI or endoscopic evaluation, thereby streamlining the diagnostic pathway and optimizing resource utilization. Consequently, the proposed biomarker panel may assist in differentiating PFCD from CAF and guide individualized therapeutic strategies.

## Supplementary Information

Below is the link to the electronic supplementary material.

Supplementary Material 1

Supplementary Material 2

Supplementary Material 3

## Data Availability

The data that support the findings of this research can be obtained from the corresponding author upon reasonable request.
