# Supplementary material for: Metabolic profiles of amino acids in patients with crohn’s disease-associated perianal fistulas and cryptoglandular anal fistulas
Source: Sci Rep. 2026 Jan 19;16:3366. doi: 10.1038/s41598-025-33334-7 (PMC12835150; doi:10.1038/s41598-025-33334-7)
Supplement: Supplementary file 2 — Supplementary Material 2 [file 41598_2025_33334_MOESM2_ESM.pdf]

KEGG Database Project
Kanehisa Laboratories
www.kanehisa.jp

Ref: 253141

Permission is granted to Scientific Reports of Springer Nature Ltd to publish both in print and digital under the CC BY 4.0 open access license the result of using KEGG and the following KEGG images in the article "Metabolic profiles of amino acids in patients with Crohn's disease-associated perianal fistulas and cryptoglandular anal fistulas" written by Kai Ma, Cong Hu, Yi Fu, Yu Liu, Feiyang Weng, Yibo Yao, Chen Wang and colleagues:

- Alanine, aspartate and glutamate metabolism (map00250)
- Arginine biosynthesis (map00220)
- Histidine metabolism (map00340)
- Phenylalanine, tyrosine and tryptophan biosynthesis (map00400)
- Phenylalanine metabolism (map00360)

subject to the condition that the original source is acknowledged by citing at least one KEGG paper.

Permission granted: Naomi Takahashi

Naomi Takahashi, Kanehisa Laboratories

Date: 9 September 2025

Copyright holder: Kanehisa Laboratories
