# Supplementary material for: Metabolic profiles of amino acids in patients with crohn’s disease-associated perianal fistulas and cryptoglandular anal fistulas
Source: Sci Rep. 2026 Jan 19;16:3366. doi: 10.1038/s41598-025-33334-7 (PMC12835150; doi:10.1038/s41598-025-33334-7)
Supplement: Supplementary file 3 — Supplementary Material 3 [file 41598_2025_33334_MOESM3_ESM.docx]

**Table S1** The calibration curve parameters of 25 analytical amino acids

| Analyte | IS | Slope | Intercept | R^2^ | Range (ng/mL) |
| --- | --- | --- | --- | --- | --- |
| AIBA | Ala-D4 | 0.0138 | 0.0273 | 0.9941 | 0.1-100 |
| Ala | Ala-D4 | 0.00294 | 0.0195 | 0.9964 | 0.1-100 |
| Arg | Ala-D4 | 0.00043 | 0.00151 | 0.9963 | 0.1-100 |
| Asn | Ala-D4 | 0.00295 | 0.00192 | 0.9965 | 0.1-100 |
| Asp | Glu-D5 | 0.00353 | 0.00561 | 0.9964 | 0.1-100 |
| Cit | Glu-D5 | 0.0167 | 0.0375 | 0.998 | 0.1-100 |
| Cys | Ala-D4 | 0.0005 | -0.0979 | 0.9988 | 0.5-500 |
| GIn | Ala-D4 | 0.0125 | 0.0177 | 0.9971 | 0.1-100 |
| Glu | Glu-D5 | 0.0117 | 0.0125 | 0.9953 | 0.1-100 |
| Gly | Ala-D4 | 0.000311 | 0.00146 | 0.997 | 0.1-100 |
| His | Ala-D4 | 0.00339 | -0.00286 | 0.9949 | 0.5-500 |
| hPro | Ala-D4 | 0.00828 | 0.0137 | 0.9973 | 0.1-100 |
| Leu/Ile | Ala-D4 | 0.00201 | -0.000383 | 0.9967 | 0.1-100 |
| Lys | Ala-D4 | 0.00345 | -0.0199 | 0.996 | 0.5-500 |
| Met | Ala-D4 | 0.0079 | 0.00115 | 0.9966 | 0.1-100 |
| Orn | Ala-D4 | 0.00188 | 0.00391 | 0.9955 | 0.1-100 |
| Phe | Phe-D5 | 0.00692 | 0.023 | 0.9978 | 0.1-100 |
| Pro | Ala-D4 | 0.0163 | 0.00367 | 0.9951 | 0.1-100 |
| Sar | Ala-D4 | 0.00354 | 0.00822 | 0.9941 | 0.1-100 |
| Ser | Ser-D3 | 0.00419 | 0.0374 | 0.9937 | 0.1-100 |
| Thr | Ala-D4 | 0.00616 | 0.0123 | 0.998 | 0.1-100 |
| Trp | Trp-D5 | 0.00726 | -0.0000277 | 0.9982 | 0.1-100 |
| Tyr | Ala-D4 | 0.00767 | 0.00216 | 0.9948 | 0.1-100 |
| Val | Ala-D4 | 0.0171 | 0.0165 | 0.9961 | 0.1-100 |

**Table S2** Intra-day and inter-day accuracy and precision of amino acids

| Analytes | Intra-day (*n* = 6) | | | | | | | | Inter-day (*n* = 18) | | | | | | | |
| --- | --- | --- | --- | --- | --- | --- | --- | --- | --- | --- | --- | --- | --- | --- | --- | --- |
|  | Accuracy (%) | | | | Precision R.S.D. (%) | | | | Accuracy (%) | | | | Precision R.S.D. (%) | | | |
|  | LLOQ | LQC | MQC | HQC | LLOQ | LQC | MQC | HQC | LLOQ | LQC | MQC | HQC | LLOQ | LQC | MQC | HQC |
| AIBA | 90.1-96.9 | 95.9-99.9 | 95.7-99.4 | 93.3-103.1 | 9.7-11.6 | 8.6-9.6 | 7.6-9.0 | 2.4-8.1 | 93.0 | 98.5 | 97.4 | 98.2 | 10.6 | 8.8 | 7.8 | 7.2 |
| Ala | 86.9-96.5 | 94.6-103.6 | 95.6-97.9 | 96.3-99.6 | 6.8-8.5 | 7.5-9.7 | 6.5-7.2 | 4.8-8.9 | 92.9 | 98.8 | 96.8 | 98.2 | 8.7 | 8.8 | 6.5 | 6.7 |
| Arg | 90.5-98.2 | 93.2-99.4 | 98.3-104.8 | 95.1-102.5 | 7.6-10.9 | 5.9-10.0 | 3.9-6.9 | 4.0-7.2 | 94.8 | 95.5 | 102.0 | 99.5 | 9.7 | 8.7 | 6.3 | 6.6 |
| Asn | 92.2-101.3 | 94.1-100.8 | 98.9-103.7 | 98.0-102.6 | 4.6-8.9 | 8.1-8.8 | 4.3-6.3 | 5.9-8.5 | 96.6 | 97.9 | 101.0 | 100.2 | 8.2 | 8.5 | 5.4 | 6.9 |
| Asp | 87.7-98.4 | 95.4-97.9 | 96.3-104.2 | 97.6-103.3 | 5.4-10.6 | 5.5-9.5 | 3.8-9.8 | 3.3-7.5 | 93.2 | 96.3 | 100.6 | 100.0 | 10.0 | 7.2 | 7.8 | 5.8 |
| Cit | 91.0-99.9 | 95.5-103.0 | 98.0-104.7 | 98.6-104.8 | 9.1-13.9 | 9.2-10.1 | 5.3-6.9 | 2.7-8.5 | 94.1 | 98.6 | 102.3 | 101.2 | 11.2 | 9.7 | 6.6 | 6.3 |
| Cys | 94.6-101.2 | 98.2-100.8 | 97.7-101.0 | 98.1-102.0 | 7.5-14.0 | 5.9-7.0 | 6.5-8.5 | 5.4-7.5 | 98.7 | 99.2 | 99.2 | 100.5 | 10.2 | 6.3 | 7.5 | 6.5 |
| GIn | 102.6-108.0 | 95.5-110.1 | 99.3-102.5 | 95.0-102.6 | 4.9-11.8 | 2.9-7.9 | 7.2-8.4 | 5.0-7.7 | 105.0 | 103.7 | 100.4 | 100.0 | 8.2 | 8.5 | 7.3 | 7.2 |
| Glu | 88.7-94.2 | 93.6-103.1 | 98.2-101.2 | 99.2-101.3 | 4.6-9.7 | 4.6-8.1 | 5.1-7.8 | 5.5-8.0 | 91.9 | 99.4 | 99.7 | 100.1 | 7.6 | 7.9 | 6.5 | 6.2 |
| Gly | 95.9-98.7 | 101.2-109.4 | 98.4-102.3 | 96.7-102.1 | 7.4-10.4 | 3.8-9.6 | 4.2-7.1 | 5.2-5.8 | 97.0 | 104.8 | 100.3 | 99.8 | 8.5 | 7.1 | 5.7 | 5.8 |
| His | 103.3-106.5 | 99.3-103.2 | 97.6-102.4 | 96.1-102.3 | 5.4-10.3 | 7.4-8.4 | 4.5-8.2 | 4.9-6.9 | 104.9 | 101.1 | 99.4 | 98.6 | 8.4 | 7.7 | 6.4 | 6.0 |
| hPro | 95.2-103.0 | 96.1-102.6 | 99.1-103.7 | 99.5-101.5 | 11.2-12.6 | 6.1-8.2 | 4.4-7.4 | 4.6-6.6 | 99.7 | 100.4 | 101.2 | 100.3 | 11.9 | 7.5 | 6.4 | 5.3 |
| Leu/Ile | 97.0-100.8 | 95.9-101.6 | 93.6-101.8 | 95.7-100.0 | 6.4-11.3 | 4.5-9.3 | 6.1-8.5 | 6.1-6.7 | 98.4 | 98.8 | 97.8 | 97.7 | 8.9 | 7.6 | 7.8 | 6.3 |
| Lys | 99.4-107.0 | 100.7-106.0 | 95.4-100.8 | 98.5-103.8 | 7.4-12.1 | 4.8-8.7 | 3.7-6.0 | 6.7-7.9 | 103.3 | 102.7 | 98.4 | 100.8 | 9.4 | 7.1 | 5.3 | 7.2 |
| Met | 94.8-100.3 | 97.5-108.0 | 96.0-96.9 | 99.0-103.3 | 5.2-10.5 | 5.0-7.3 | 4.4-9.4 | 4.5-6.4 | 98.4 | 102.6 | 96.5 | 100.6 | 8.0 | 7.0 | 6.5 | 5.4 |
| Orn | 88.3-101.8 | 97.3-103.1 | 101.5-103.6 | 98.6-100.8 | 5.9-9.7 | 6.6-8.9 | 4.5-7.3 | 4.7-6.5 | 95.9 | 100.6 | 102.8 | 99.4 | 9.8 | 7.5 | 5.8 | 5.3 |
| Phe | 95.4-97.6 | 96.6-100.8 | 100.7-103.7 | 98.9-99.0 | 7.8-12.5 | 6.1-12.1 | 5.9-10.5 | 4.4-6.4 | 96.8 | 99.3 | 102.4 | 98.9 | 10.1 | 8.4 | 7.9 | 5.2 |
| Pro | 93.7-100.0 | 98.3-108.6 | 95.6-102.6 | 94.7-101.6 | 10.3-13.1 | 6.0-10.6 | 6.1-8.8 | 5.8-6.3 | 96.6 | 102.9 | 99.4 | 98.4 | 11.0 | 9.2 | 7.9 | 6.4 |
| Sar | 94.5-98.9 | 96.4-100.7 | 97.3-105.1 | 95.0-103.2 | 3.7-11.0 | 9.7-9.9 | 3.1-8.7 | 4.5-7.1 | 96.1 | 99.1 | 100.7 | 99.8 | 7.4 | 9.4 | 6.7 | 6.4 |
| Ser | 90.2-93.9 | 98.7-101.4 | 96.8-100.4 | 94.8-101.9 | 4.8-13.2 | 7.5-10.0 | 4.9-9.4 | 3.7-5.8 | 92.5 | 100.4 | 99.0 | 98.9 | 9.0 | 8.5 | 7.3 | 5.5 |
| Thr | 94.5-97.8 | 96.4-99.5 | 98.7-103.0 | 98.4-105.6 | 6.6-9.7 | 6.5-10.3 | 4.2-8.2 | 3.5-6.0 | 96.2 | 98.5 | 100.8 | 101.7 | 7.6 | 8.7 | 6.5 | 5.7 |
| Trp | 93.2-99.4 | 97.7-101.9 | 99.1-103.8 | 95.8-99.4 | 8.0-9.8 | 3.2-7.3 | 4.3-7.4 | 5.1-6.1 | 96.1 | 100.3 | 100.8 | 97.8 | 9.0 | 6.0 | 6.0 | 5.6 |
| Tyr | 93.3-96.4 | 98.7-105.3 | 97.1-102.3 | 97.3-102.2 | 7.2-12.8 | 6.0-7.5 | 8.4-9.8 | 3.7-7.7 | 94.3 | 101.8 | 100.2 | 99.0 | 9.1 | 6.8 | 8.8 | 6.3 |
| Val | 91.6-96.5 | 98.3-104.4 | 94.0-103.3 | 94.8-103.0 | 10.9-12.6 | 6.1-8.9 | 5.1-6.7 | 5.4-8.8 | 93.6 | 101.7 | 98.2 | 99.5 | 11.3 | 7.7 | 7.1 | 7.2 |

**Table S3** Extraction recoveries and matrix effects of amino acids in serum (*n* = 6)

| Analytes | Extraction Recovery | | | | | | Matrix effect | | | | | |
| --- | --- | --- | --- | --- | --- | --- | --- | --- | --- | --- | --- | --- |
|  | LQC | | MQC | | HQC | | LQC | | MQC | | HQC | |
|  | Mean extraction recovery | R.S.D. (%) | Mean extraction recovery | R.S.D. (%) | Mean extraction recovery | R.S.D. (%) | Mean extraction recovery | R.S.D. (%) | Mean extraction recovery | R.S.D. (%) | Mean extraction recovery | R.S.D. (%) |
| AIBA | 99.1 | 9.6 | 99.5 | 9.0 | 99.0 | 8.1 | 100.8 | 10.5 | 92.6 | 5.9 | 97.9 | 5.1 |
| Ala | 98.9 | 9.7 | 97.9 | 6.9 | 105.0 | 8.9 | 99.2 | 12.6 | 94.8 | 7.3 | 92.4 | 8.3 |
| Arg | 102.8 | 9.4 | 106.3 | 3.9 | 96.0 | 7.2 | 96.3 | 9.4 | 92.5 | 10.2 | 96.1 | 6.5 |
| Asn | 94.4 | 8.5 | 104.7 | 5.0 | 96.0 | 6.5 | 97.4 | 3.5 | 92.0 | 7.3 | 98.7 | 3.1 |
| Asp | 99.9 | 5.5 | 106.5 | 9.8 | 96.7 | 7.5 | 95.4 | 7.7 | 92.0 | 8.5 | 105.6 | 6.6 |
| Cit | 100.7 | 10.1 | 104.5 | 6.3 | 102.1 | 8.5 | 98.0 | 4.9 | 91.9 | 6.4 | 96.2 | 7.1 |
| Cys | 97.5 | 5.9 | 104.3 | 6.5 | 103.2 | 6.9 | 94.4 | 10.0 | 91.8 | 7.6 | 96.8 | 8.4 |
| GIn | 109.9 | 2.9 | 99.7 | 7.3 | 98.7 | 7.7 | 97.9 | 9.2 | 92.9 | 8.1 | 106.4 | 8.0 |
| Glu | 107.1 | 4.6 | 101.3 | 7.3 | 100.5 | 5.5 | 95.7 | 10.9 | 96.3 | 7.3 | 95.5 | 8.7 |
| Gly | 107.4 | 3.8 | 96.4 | 5.8 | 101.6 | 5.8 | 96.2 | 8.8 | 97.4 | 5.0 | 98.4 | 10.1 |
| His | 98.0 | 8.3 | 102.6 | 4.5 | 101.1 | 4.9 | 98.2 | 5.3 | 95.7 | 3.2 | 95.6 | 8.4 |
| hPro | 97.4 | 8.2 | 107.3 | 4.4 | 101.6 | 5.1 | 96.6 | 6.7 | 91.1 | 10.8 | 99.9 | 8.9 |
| Leu/Ile | 93.2 | 8.7 | 98.6 | 7.3 | 103.4 | 6.4 | 99.3 | 8.7 | 101.3 | 7.3 | 94.0 | 6.4 |
| Lys | 98.9 | 7.6 | 99.5 | 6.0 | 107.2 | 7.1 | 98.5 | 9.2 | 98.3 | 9.0 | 97.7 | 6.2 |
| Met | 102.7 | 5.0 | 93.4 | 4.4 | 105.3 | 5.0 | 103.4 | 6.0 | 100.6 | 5.6 | 94.7 | 6.8 |
| Orn | 100.4 | 7.0 | 107.2 | 7.3 | 103.4 | 4.7 | 99.5 | 9.8 | 93.9 | 9.6 | 92.6 | 6.7 |
| Phe | 102.8 | 6.1 | 104.8 | 10.5 | 106.1 | 5.5 | 99.2 | 9.4 | 91.1 | 7.7 | 89.3 | 7.4 |
| Pro | 101.6 | 6.0 | 101.4 | 8.3 | 95.6 | 6.0 | 95.8 | 6.3 | 97.8 | 8.1 | 100.6 | 9.7 |
| Sar | 101.0 | 9.9 | 100.2 | 8.7 | 93.5 | 4.8 | 98.7 | 9.0 | 91.5 | 5.7 | 105.8 | 8.0 |
| Ser | 103.1 | 10.0 | 101.9 | 9.4 | 92.1 | 3.7 | 98.2 | 10.8 | 95.7 | 7.5 | 97.4 | 8.9 |
| Thr | 94.9 | 10.0 | 105.6 | 7.1 | 106.6 | 6.0 | 103.4 | 9.0 | 91.6 | 8.3 | 92.1 | 8.0 |
| Trp | 94.4 | 7.3 | 103.2 | 7.4 | 102.5 | 6.1 | 107.2 | 6.8 | 94.6 | 8.8 | 94.8 | 9.2 |
| Tyr | 93.3 | 6.0 | 99.6 | 9.8 | 100.3 | 7.7 | 105.6 | 10.3 | 98.9 | 4.4 | 100.1 | 6.9 |
| Val | 102.2 | 8.9 | 102.0 | 6.7 | 97.4 | 8.8 | 94.2 | 8.9 | 100.2 | 9.3 | 97.0 | 5.4 |

**Table S4 Sample stability of AAs in Serum (*n* = 6)**

| Analytes | 3 h at room temperature | | | | | | Three freeze-thaw cycles | | | | | |
| --- | --- | --- | --- | --- | --- | --- | --- | --- | --- | --- | --- | --- |
|  | LQC | | MQC | | HQC | | LQC | | MQC | | HQC | |
|  | Accuracy (%) | R.S.D. (%) | Accuracy (%) | R.S.D. (%) | Accuracy (%) | R.S.D. (%) | Accuracy (%) | R.S.D. (%) | Accuracy (%) | R.S.D. (%) | Accuracy (%) | R.S.D. (%) |
| AIBA | 98.2 | 6.9 | 98.5 | 9.6 | 99.3 | 7.5 | 99.5 | 11.9 | 100.1 | 7.2 | 95.9 | 7.3 |
| Ala | 95.2 | 10.0 | 101.2 | 8.1 | 96.7 | 6.4 | 106.5 | 4.4 | 101.5 | 7.1 | 102.4 | 5.2 |
| Arg | 100.0 | 8.8 | 96.6 | 9.8 | 96.3 | 8.6 | 103.5 | 5.2 | 101.8 | 7.8 | 100.1 | 6.3 |
| Asn | 97.3 | 10.8 | 102.3 | 9.3 | 99.8 | 9.1 | 102.6 | 10.7 | 99.5 | 6.8 | 100.6 | 7.0 |
| Asp | 98.5 | 9.7 | 101.1 | 7.5 | 98.1 | 8.0 | 103.1 | 10.0 | 99.8 | 6.3 | 96.0 | 6.4 |
| Cit | 102.3 | 7.8 | 96.0 | 7.7 | 98.1 | 8.5 | 93.9 | 7.2 | 102.8 | 7.3 | 98.6 | 6.0 |
| Cys | 103.1 | 6.6 | 102.8 | 4.7 | 99.1 | 5.6 | 104.6 | 5.4 | 97.9 | 6.5 | 96.8 | 5.7 |
| GIn | 96.8 | 9.6 | 97.9 | 5.4 | 98.7 | 5.2 | 98.8 | 9.7 | 97.1 | 7.0 | 101.3 | 6.9 |
| Glu | 99.5 | 11.0 | 99.6 | 7.4 | 102.4 | 6.2 | 99.4 | 7.9 | 100.2 | 6.6 | 99.1 | 5.2 |
| Gly | 104.2 | 9.1 | 102.6 | 6.3 | 100.4 | 6.8 | 93.1 | 11.0 | 101.3 | 7.7 | 102.7 | 3.8 |
| His | 100.5 | 7.2 | 100.8 | 4.0 | 99.7 | 3.9 | 104.4 | 2.7 | 97.7 | 6.0 | 97.4 | 3.5 |
| hPro | 95.9 | 4.8 | 101.5 | 5.1 | 101.1 | 7.0 | 101.2 | 9.7 | 104.2 | 5.6 | 101.0 | 6.4 |
| Leu/Ile | 98.8 | 7.9 | 98.5 | 6.9 | 96.4 | 4.0 | 102.3 | 6.7 | 101.3 | 5.7 | 100.5 | 6.5 |
| Lys | 102.4 | 10.8 | 100.4 | 6.2 | 95.2 | 7.6 | 98.9 | 8.5 | 101.7 | 7.9 | 96.9 | 4.0 |
| Met | 100.3 | 7.4 | 99.5 | 10.8 | 99.6 | 7.9 | 98.6 | 4.2 | 100.1 | 6.3 | 101.5 | 6.0 |
| Orn | 97.2 | 4.6 | 99.8 | 7.9 | 96.7 | 9.3 | 100.3 | 6.6 | 97.2 | 5.1 | 96.8 | 5.0 |
| Phe | 99.2 | 8.5 | 98.0 | 7.3 | 94.9 | 7.2 | 95.3 | 4.7 | 100.9 | 9.4 | 108.4 | 1.4 |
| Pro | 95.7 | 7.1 | 98.3 | 6.3 | 98.8 | 10.3 | 94.5 | 6.0 | 104.8 | 6.6 | 99.8 | 7.6 |
| Sar | 101.3 | 8.9 | 100.5 | 9.6 | 96.7 | 6.8 | 102.6 | 7.2 | 101.3 | 7.7 | 103.3 | 6.1 |
| Ser | 100.6 | 9.3 | 106.4 | 4.8 | 101.6 | 7.4 | 95.2 | 7.7 | 99.0 | 8.0 | 102.9 | 6.4 |
| Thr | 102.8 | 10.3 | 100.4 | 7.2 | 101.9 | 6.0 | 101.2 | 4.9 | 97.9 | 6.9 | 99.4 | 5.9 |
| Trp | 104.2 | 8.4 | 104.1 | 9.0 | 99.5 | 9.2 | 98.2 | 6.5 | 102.5 | 6.5 | 105.5 | 5.4 |
| Tyr | 106.3 | 6.5 | 105.3 | 5.2 | 100.8 | 7.1 | 101.0 | 8.1 | 104.7 | 4.6 | 99.8 | 6.1 |
| Val | 99.5 | 4.2 | 103.5 | 7.1 | 100.6 | 6.6 | 102.0 | 8.4 | 103.3 | 5.2 | 99.7 | 7.4 |
